# The association between geographic proximity to a dialysis facility and use of dialysis catheters

**DOI:** 10.1186/1471-2369-15-40

**Published:** 2014-02-27

**Authors:** Lisa M Miller, Lavern M Vercaigne, Louise Moist, Charmaine E Lok, Navdeep Tangri, Paul Komenda, Claudio Rigatto, Julie Mojica, Manish M Sood

**Affiliations:** 1 Health Science Centre, Winnipeg, MB, Canada; 2 Faculty of Pharmacy, Apotex Building, Winnipeg, MB, Canada; 3 Department of Medicine, Division of Nephrology, University of Western Ontario London, ON, Canada; 4 Department of Medicine, Section of Nephrology, University Health network and the University of Toronto, Toronto, ON, Canada; 5 Seven Oaks Hospital, Winnipeg, MB, Canada; 6 University of Manitoba, Winnipeg, MB, Canada; 7 Ottawa Hospital Research Institute, Section of Nephrology, The Ottawa Hospital, Ottawa, ON, Canada

**Keywords:** Vascular access, Central venous catheters, Arteriovenous fistula, Remote community, Rural community

## Abstract

**Background:**

Residing remotely from health care resources appears to impact quality of care delivery. It remains unclear if there are differences in vascular access based on distance of one’s residence to dialysis centre at time of dialysis initiation, and whether region or duration of pre-dialysis care are important effect modifiers.

**Methods:**

We studied the association of distance from a patients’ residence to the nearest dialysis centre and central venous catheter (CVC) use in an observational study of 26,449 incident adult dialysis patients registered in the Canadian Organ Replacement Registry between 2000–2009. Multivariate logistic regression was used to assess the association between distance in tertiles and CVC use, adjusted for patient demographics and comorbidities. Geographic region and duration of pre-dialysis care were examined as potential effect modifiers.

**Results:**

Eighty percent of patients commenced dialysis with a CVC. Incident CVC use was highest among those living > 20 km from the dialysis centre (OR 1.29 (1.24-1.34)) compared to those living < 5 km from centre. The length of pre-dialysis care and geographic region were significant effect modifiers; among patients residing in the furthest tertile (>20 km) from the nearest dialysis centre, incident CVC use was more common with shorter length of pre-dialysis care (< 1 year) and residence in central regions of the country.

**Conclusion:**

Residing further from a dialysis centre is associated with increased CVC use, an effect modified by shorter pre-dialysis care and the geographic region of the country. Efforts to reduce geographical disparities in pre dialysis care may decrease CVC use.

## Background

Vascular access is a major contributor to overall morbidity and mortality in the hemodialysis population [1-5]. Central venous catheters (CVCs) are increasingly used as the first-line vascular access in incident hemodialysis patients [6,7]. Mortality risk in those commencing hemodialysis with a CVC has been reported as six times greater than the risk of death in arteriovenous fistula (AVF) or arteriovenous graft (AVG) use combined [8]. Evidence informed guidelines statements suggest that a functioning reliable AVF is the preferred vascular access because it has the lowest rate of infection, access thrombosis, salvage interventions, and mortality compared with the CVC and AVG [1-5,8-11].

Many patients with chronic kidney disease (CKD) and end stage renal disease (ESRD) live in rural and remote communities, where there are no dialysis centres or local access to nephrology and surgical care. In the general population, there is growing evidence supporting an association between residence location and access to care, where rural residents receive less optimal health care and have overall poorer health compared to urban residents [12-17]. Less is known about the CKD and ESRD population residing in rural and remote locations; however, an association with increased mortality and poor achievement of evidence-based quality indicators has been shown [18-20]. Areas for intervention and improvement in rural populations are often difficult to identify, costly and resource intensive [21,22].

Initiation of hemodialysis with a functioning AVF requires multiple steps, including timely nephrology and surgical assessment and access creation with sufficient time for maturation and/or facilitative intervention as required. Furthermore the likelihood of dialysis initiation with an AVF varies based on the geographic region of the country and the presence and duration of pre-dialysis care [23,24]. Residing remotely may negatively impact this necessary sequence of events, often resulting in the need for dialysis initiation with a CVC. This study examines the association between distance from patients’ residence to the nearest dialysis treatment centre and incident CVC use, and whether the length of pre-dialysis care and geographic region were effect modifiers.

## Methods

### Study design

This study was approved by the Research Board and the Hospital Ethics Board at St. Boniface Hospital in Winnipeg, Manitoba. All adults (>18 years old) who initiated chronic hemodialysis with a documented vascular access type and registered in the Canadian Organ Replacement Registry (CORR) between January 1, 2000 and December 2009 (excluding Quebec) were included in our analysis and followed until April 2011. Patients with less than 90 days follow up from dialysis initiation were excluded. Residents of Quebec were excluded because special permissions to use their data were not obtained. The CORR is a validated national registry that records the incidence, prevalence and outcome of all chronic dialysis and solid organ transplant patients in Canada [25]. Data including demographics, comorbidities, dialysis modality, vascular access status, transplantation, and death is prospectively collected by voluntary completion of survey forms for each patient at dialysis initiation and updated annually on October 31 of each year.

### Definitions

Incident vascular access was defined as the access used at the first hemodialysis treatment and categorized as either CVC or AVF/G. Grafts encompassed < 5% of total vascular accesses, and were included as one category (AVF/G). CVCs included both tunneled cuffed and temporary CVCs. Patients using a CVC with a maturing AVF/G were included in the CVC group.

Distance to centre was calculated as the direct linear distance in kilometers (km) between a patient’s postal code of their primary residence at dialysis initiation to the nearest dialysis centre using Vincenty’s formula, a validated iterative method to calculate the distance between two points, while accounting for the Earth’s curvature [26]. Distance was divided into tertiles < 5 km, 5–20 km, and > 20 km. Pre-dialysis care was defined as whether or not patients had their first contact with a nephrologist > 90 days prior to the initiation of hemodialysis therapy. Duration of pre-dialysis care was the time between first contact with a nephrologist and initiation of first dialysis. This information is collected on the CORR data collection form at time of initiation of dialysis. Race was based on patients’ self report. Co-morbid illnesses included a history of angina, myocardial infarction, coronary artery bypass surgery, diabetes mellitus, peripheral vascular disease, malignancy, hypertension, cigarette smoking, lung disease, cerebral vascular disease (CVD), and any serious illness. Comorbidities and laboratory data were ascertained at the start of dialysis. Causes of ESRD included hypertension, diabetes mellitus, glomerulonephritis, interstitial disease, polycystic kidney disease, obstruction, other and unknown. Provinces and territories were categorized as geographic regions as follows: Atlantic (New Brunswick, Nova Scotia, Prince Edward Island, Newfoundland), Central (Ontario), Prairies (Alberta, Saskatchewan, Manitoba, Nunavut, Northwest Territories), Pacific (British Columbia, Yukon).

### Outcome measures

The outcome of interest was incident CVC usage in the hemodialysis cohort. Incident access was determined at dialysis initiation.

### Statistical analyses

Continuous variables were summarized as means or medians with standard deviation or inter-quartile ranges, respectively depending on their data distribution. Differences in baseline characteristics between distant cohorts were determined by the Z test, students *t*-test or the Kruskal Wallis test for continuous variables and chi-square or the Mann–Whitney test for dichotomous variables, according to the data distribution.

To assess our outcome of incident CVC usage, we created separate multivariate logistic regression models adjusted for the distance from centre, demographics such as age, sex, race, dialysis era, pre-dialysis care, geographic region, comorbidities, BMI, cause of ESRD and initial laboratory investigations (serum albumin, hemoglobin). Distance from the centre was modeled as a continuous variable but categorized into tertiles as < 5, 5–20 and > 20 km from the closest dialysis centre. Formal interaction terms were examined for effect modification.

To examine the relationship between pre-dialysis care and distance to centre, the interaction of distance to centre and the binary presence/absence of pre-dialysis care was first determined. The analysis was then limited to those who received pre-dialysis care (nephrologist contact > 90 days before dialysis initiation) and categorized by duration of care < 1, 1–3 and > 3 years. Similarly the impact of distance to centre and incident vascular access based on geographic regions was examined by a formal interaction term of region and distance.

Multiple imputation [27] was employed for missing values for BMI (9.2%), pre-dialysis care (17%), etiology of ESRD (3.7%), co-morbidities(2.3%), hemoglobin(8.3%), and albumin(15.3%) with a random draw from the predictive distribution of an imputation model including age, sex, and race, repeated ten times. An iterative Markov chain Monte Carlo (MCMC) method was used. Separate models were created using original and imputed data, and as no statistically significant differences were noted, pooled estimates of 10 iterations were reported.

Analyses were performed using PASW Version 18. All hypothesis tests were two sided with statistical significance defined as having a P value of <0.05.

## Results

### Patient characteristics stratified by distance from nearest dialysis provider

The original study cohort consisted of 29,406 patients with known vascular access (96% of all incident patients), but 2378 were excluded due to follow-up < 90 days after initiation of dialysis, and 579 were excluded due to missing distance data. Our study cohort consisted of 26, 449 incident hemodialysis patients with known vascular access (CVC 79.8%, AVF 20.2%) at initiation of dialysis between January 2001 and December 2009 in the CORR database. Female sex, co-morbid illnesses (diabetes, myocardial infarction, CABG, pulmonary edema, malignancy, any serious illness) were associated with incident CVC use whereas increasing age, hemoglobin, albumin, BMI and hypertension were associated with incident AVF/AVG (data not shown). Patients residing > 20 km from a dialysis centre were more likely to be younger, either Caucasian or Aboriginal, have a higher BMI and reside in the Atlantic or Prairie provinces (Table 1). There were no substantive differences in co-morbidities based on distance, however less ESRD due to hypertension was seen in those residing > 20 km. Mean albumin, mean hemoglobin, the proportion receiving pre-dialysis care and the duration of pre-dialysis care were all lower in those residing > 20 km. Although rates of CVC use at start of dialysis were high overall, patients who resided > 20 km from the nearest dialysis centre had higher CVC usage (82.3%) than patients who lived < 5 km (79.7%) and 5–20 km (78.1%) (Table 1).

**Table 1:** Baseline characteristics by distance from residence postal code and nearest dialysis facility (% or mean ± SD)

	**< 5 km**	**5-20 km**	**>20 km**
	**% (N)**	**% (N)**	**% (N)**
**N**	27.2(7192)	35.1(9287)	37.7(9970)
CVC*	79.7 (5659)a	78.1(7251)a	82.3(8202)b
AVF/AVG**	21.3(1533)a	21.9(2036)a	17.7(1768)b
**Age p < 0.001**	67.5(14.9)	66.2(15.3)	64.8(15.0)
**Sex (% female)**	41.9(3012)a	40.3(3740)b	40.4(4027)a,b
**Race**			
**Caucasian**	69.7(5014)a	68.1(6324)a	78.0(7780)b
**Aboriginal**	4.9(349)a	1.8(170)b	11.4(1139)c
**East Asian**	7.4(534)a	9.1(842)b	1.8(182)c
**South Asian**	9.9(710)a	11.9(1101)b	6.5(649)c
**Black**	4.0(297)a	4.8(448)b	1.1(108)c
**Other**	4.0(288)a	4.3(402)a	1.1(112)b
**BMI p < 0.0001**	27.4(7.0)	27.7(7.1)	28.1(7.1)
**Geographic location**			
**Atlantic**	21.8(609)a	22.3(622)b	55.9(1560)c
**Central**	29.1(4196)a	38.3(5462)a	32.6(4647)b
**Prairie**	24.9(1495)a	32.6(1955)a	42.5(2550)b
**Pacific**	27.7(942)ab	36.7(1248)b	35.6(1213)a
**Co-morbidities**			
**Angina**	22.4(1613)a	21.7(2015)a	21.9(2180)a
**Acute coronary syndrome**	21.6(1555)a	21.0(1954)a	22.2(2209)a
**Pulmonary edema**	27.0(1940)a	24.7(2298)b	27.5(2738)b
**Diabetes mellitus**	48.7(3500)a	46.3(4299)b	48.1(4792)a
**Stroke**	15.1(1087)a	13.5(1253)b	14.3(1421)b
**Peripheral vascular disease**	19.2(1380)a	17.5(986)b	12.4(1238)b
**Malignancy**	11.5(828)b	10.6(1149)a	12.7(1264)a
**Lung disease**	12.1(924)a	10.9(1180)b	12.9(1178)a
**Hypertension**	83.1(5977)a	82.5(7665)a,b	82.3(8207)b
**Serious Illness**	11.2(809)a	11.1(1034)a	10.4(1036)a
**Current smoker**	13.3(959)a	11.0(1017)b	15.6(1557)c
**CABG**	13.3(954)a	13.8(1282)a	13.6(1355)a
**Cause of ESRD:**			
**Hypertension**	23.9(1718)a	22.9(2476)a	21(1918)b
**Diabetes mellitus**	39.0(2807)a	36.1(3909)b	38.4(3514)a
**Glomerulonephritis**	12.6(906)a	14.9(1611)b	14.8(1355)b
**Obstruction**	3.0(213)a	3.8(409)b	3.9(354)b
**Interstitial disease**	0.9 (63)a	1.0 (91)a	1.1(107)a
**Polycystic kidney disease**	3.0(216)a	3.9(364)a	4.1(404)a
**Other**	7.0(504)a	7.7(713)a,b	8.1(808)b
**Unknown**	10.6(765)a	9.7(905)a	8.9(883)b
**Albumin g/L (mean ± SD) p < 0.0001**	32.3(11.9)	32.0(7.7)	31.4(8.8)
**Hemoglobin g/L (mean ± SD) p = 0.02**	101.1(20.5)	100.8(18.4)	99.9(33.3)
**Any pre-dialysis care (>90 days)**	58.9(4233)a	57.3(5318)b	55.7(5553)c
**Pre-HD care < 1 year+**	27.9(1652)a	34.8(2057)a	37.3(2203)a
**Pre-HD care 1–3 years+**	28.0(1561)a	35.6(1984)a	36.3(2023)a
**Pre-HD care > 3 years+**	28.1(1471)a	35.4(1853)a	36.5(1910)a

Similar letters denote a subset of distance categories whose column proportions do not differ significantly from each other at the 0.05 level.

* CVC – central venous catheter.

** AVF/AVG – Arteriovenous fistula or arteriovenous graft.

+ limited to patients with any pre-dialysis care.

### Association of distance and incident catheter use

Distance was significantly associated with incident CVC use in adjusted models (p < 0.0001) (Table 2). In comparison to patients living within 5 km of a dialysis centre, patients living > 20 km away were almost 30% more likely to initiate dialysis with a CVC after adjustment for covariates including age, race, sex, BMI, albumin, hemoglobin, co-morbidities, region, and pre-dialysis care (OR 1.29 (95% CI 1.0-1.34)).

**Table 2:** Adjusted odds ratio of incident usage by distance from centre

**Distance from dialysis centre (km)**	**% CVC (N)**	**OR of incident CVC**
<5	78.7(5659)	Referent
5-20	78.1(7251)	1.02 (1.00-1.05)
>20	82.3(8202)	1.29 (1.24-1.34)

Distance from centre P < 0.0001 for incident CVC in adjusted models.

Incident CVC adjusted for race, sex, age, BMI, alb, Hb, co-morbidities, cause of ESRD, any pre-dialysis care, region.

### Association of vascular access, distance and pre-dialysis care

We examined whether pre-dialysis care and geographic region were effect modifiers on the relationship between vascular access and distance. Overall, patients who did not receive any pre-dialysis care were more likely to receive dialysis by a CVC. In patients without any pre-dialysis care, those residing > 20 km from the nearest dialysis centre were more than 3 times as likely to initiate dialysis with a CVC compared to those residing < 5 km (OR 3.64 (95% CI 2.62-5.07))(Table 3). Even among patients with pre-dialysis care, residing > 20 km was associated with CVC use at dialysis initiation compared to residing < 5 km (OR 2.42 (2.16-2.71)).

**Table 3:** Adjusted odds ratios of incident CVC usage by distance to centre and pre-dialysis care (distance X any pre-dialysis care interaction p < 0.0001)

**Pre-dialysis care**	**Distance from dialysis centre (km)**	**% CVC (N)**	**Adjusted OR of CVC, no pre-dialysis care**
No	<5	91.2 (2698)	Referent
	5-20	89.6 (3557)	1.02 (0.80-1.29)
	>20	89.8(3968)	3.64 (2.62-5.07)
Yes	<5	70.0 (2961)	Referent
	5-20	69.5 (3694)	1.21 (1.10-1.34)
	>20	76.2 (4234)	2.42 (2.16-2.71)

Models adjusted for race, era, sex, age, BMI, albumin, hemoglobin, co-morbidities, region, cause of ESRD.

The duration of pre-dialysis care was a significant effect modifier, where longer duration of pre-dialysis care partially attenuated the impact of distance. In patients with the shortest duration of pre-dialysis care (<1 year), those residing furthest away ( > 20 km) were more likely to commence dialysis with a CVC than those residing < 5 km (OR 1.27 (95% CI 1.05-1.54)) (Table 4). But within the patients who lived furthest away (> 20 km), incident CVC use decreased with longer pre-dialysis care (72% CVC >3 years vs 82.5% relative <1 year). Overall, patients with > 3 years pre-dialysis care residing > 20 km away from a dialysis were 30% less likely to start HD with a CVC than those with <1 year pre-dialysis care living within 5 km of a dialysis unit (OR 0.71 (0.60-0.84)).

**Table 4:** Adjusted odds ratios of incident CVC usage by distance to centre and length of pre-dialysis care (distance X length of pre-dialysis care interaction p < 0.0001)

**Length of pre-dialysis care**	**Distance from dialysis centre (km)**	**%CVC (N)**	**OR (95% CI) with length of pre-dialysis care as separate strata**	**OR (95% CI) with common referent**
< 1 year	<5	78.4 (941)	Referent	Referent
	5-20 (1)	75.6 (1119)	1.19 (0.95-1.48)	0.84 (0.70-1.01)
	>20	82.5 (1336)	2.65 (2.05-3.44)	1.27 (1.05-1.54)
1-3 years	<5	68.6 (1071)	referent	0.58 (0.49-0.70)
	5-20	70.2 (1393)	1.24 (1.05-1.46)	0.63 (0.53-0.75)
	>20	75.2 (1522)	2.52 (2.09-3.04)	0.82 (0.69-0.98)
> 3	<5	64.5 (949)	Referent	0.48 (0.40-0.57)
	5-20	63.8 (1182)	1.21 (1.03-1.41)	0.47 (0.40-0.56)
	>20	72 (1376)	2.31 (1.93-2.75)	0.71 (0.60-0.84)

Models adjusted for race, era, sex, age, BMI, albumin, hemoglobin, co-morbidities, region, cause of ESRD.

The relationship between CVC use and distance from dialysis centre varied across regions in Canada (distance X geographic region p value = 0.002) (Table 5). Residing remotely strongly increased the likelihood of initiating with a CVC in the Atlantic and Central regions of Canada; this effect was attenuated or absent in the Prairies or Pacific regions. This was especially marked in the central region (Central OR of CVC 1.31 (95% CI 1.03-1.67), p < 0.0001 and 1.93 (95% CI 1.55-2.4), p < 0.001, respectively, referent Atlantic region < 5 km).

**Table 5:** Adjusted odds ratios of incident CVC usage by distance to centre and geographic region (distance X region interaction p = 0.02)

**Region**	**Distance from dialysis centre (km)**	**% CVC (N)**	**OR 95% CI**
Atlantic	<5	75.4 (459)	Referent
	5-20	72.2 (449)	1.19 (0.89-1.61)
	> 20	76.4 (1192)	3.05 (2.30-4.04)
Central	<5	79.7 (3306)	Referent
	5-20	79.1 (4320)	1.11 (0.98-1.25)
	> 20	85.3 (3962)	2.78 (2.38-3.24)
Prairies	<5	78.5 (1173)	Referent
	5-20	79.1 (1547)	1.50 (1.20-1.88)
	> 20	82.1 (2094)	2.06 (1.64-2.59)
Pacific	<5	76.5 (721)	Referent
	5-20	74.9 (935)	1.10 (0.85-1.43)
	> 20	78.6 (954)	2.83 (2.06-3.90)

Adjusted for race, era, sex, age, BMI, albumin, hemoglobin, co-morbidities, pre-dialysis care, cause of ESRD.

## Discussion

Vascular access represents one of the most modifiable factors for improving dialysis adequacy and outcomes. Catheter use is associated with higher morbidity and mortality risk than AVF use. In this large, national cohort study we demonstrated that living further from a dialysis centre was independently associated with incident CVC use. Pre-dialysis care and geographic region were significant effect modifiers on the relationship between distance and CVC use. Taken together, these results highlight the negative effect of residing remotely from health care resources in optimal dialysis care, and suggest such disparity may be partially attenuated by appropriate pre-dialysis care.

Our study is the largest ever undertaken in examining the specific relationship between type of vascular access and geographic isolation, which is important because access type is such a profound risk modifier. Our data is consistent with previous reports, many Canadian studies, illustrating that increased distance, a surrogate marker for geographic isolation and limited access to health resources, is associated with increases in adverse outcomes, quality of care and mortality in CKD and ESRD populations [17-19,28-30].

In our study, the presence and duration of pre-dialysis care appears to partially mitigate the effect of location on the likelihood of initiating hemodialysis with a CVC. Among patients who resided > 20 km from a dialysis centre, those who received pre-dialysis care for < 1 year were significantly more likely to commence hemodialysis with a CVC than their counterparts who received > 1 year of care. Reduced access to pre-dialysis care in remote areas is described in other studies as well. A recent study of 404,602 patients in the US renal data registry showed that patients in the most rural regions and large metropolitan areas were less likely to receive pre-dialysis care > 6 or 12 months, and most likely to receive it if they lived in small/medium metropolitan areas [31]. Suboptimal pre-dialysis care is concerning because patients who receive late nephrology care are more likely to start hemodialysis with a CVC than those referred early to nephrology [32-36]. In addition, patients commencing dialysis without adequate duration of pre-dialysis care are more likely to be anemic, hyperphosphatemic, and malnourished than those who received longer nephrology care before initiation of dialysis [37,38]. Late nephrology care is also associated with increased morbidity and mortality [36-40]. However, the benefit of early referral is lost if the opportunity to address complications of CKD and plan for renal replacement therapy is not utilized to optimize dialysis initiation, as demonstrated by Hughes et al. in retrospective cohort of 436 hemodialysis patients [41]. In their study, 56% of all patients that were followed by nephrologists > 12 months still started hemodialysis with a CVC, in which 65% started without even an attempt at AVF/AVG creation.

Geographical region played a role in incident vascular access as patients in the Atlantic and the Central regions of Canada were more likely to initiate dialysis with a catheter if they resided > 20 km away from a dialysis centre. Our findings are consistent with emerging literature identifying regional differences in vascular access. In a large US study of 10,112 patients across 173 dialysis facilities, Tangri et al. found after case-mix adjustment, a large proportion of explainable variability regarding CVC use was dependent on the treating facility and geographic regions [25]. Many other aspects of dialysis care demonstrate regional variability. For example, recent studies have shown marked variation in cardioprotective medication prescription [42,43] and kidney transplanatation [44,45] across geographic regions in the United States that cannot be explained by patient demographics or burden of comorbidities.

There are several plausible explanations why residence location may impact vascular access including limited access to health care resources, reduced access to transportation, especially in rural communities where public transportation services may be very limited or expensive, and the time and cost commitments of extensive travel to centres located far from the home community. The need to coordinate appointments among multiple specialists such as vascular surgery, interventional radiology and nephrology may be additional barriers to timely vascular access assessment and creation. Socioeconomic status and education level may impact on vascular access decisions in these patients. In some provinces such as Manitoba and Saskatchewan, where there are large First nations population especially in the more rural regions, language barriers could potentially affect patient decisions regarding vascular access. We have also identified an area where recognition and interventions targeted at rural dwellers, such as rapid assessment clinics, may address these barriers.

Unfortunately, as highlighted by our study, vascular access outcomes overall remain fairly poor in Canada. Although mitigated to some degree by predialysis care, nearly 80% of all patients starting dialysis do so with a CVC regardless of whether they live close or far away from a dialysis centre. This trend has persisted despite increasing attention towards increasing fistula rates by such initiatives as Fistula First [46]. In their study of 436 incident patients, Hughes et al. discusses some possible explanations for catheter use at dialysis initiation, where patient related delays, acute on chronic renal failure, and surgical delays accounted for 78% of the reason [41]. Therefore, focused efforts towards these factors may be a first step in changing incident CVC use, but the findings of our study suggests there may be unique patient characteristics in the rural population that may still warrant a different approach.

The strengths of our study include a large, representative, national cohort with accurate ascertainment of exposures and outcomes. We also performed comprehensive case mix adjustment for multiple factors, including demographics, comorbidities, and laboratory variables thereby accounting for patient related factors that may impact dialysis access. Furthermore, although we used multiple imputation for missing values, separate models were created using original and imputed data, and no significant differences were seen. Lastly, although we chose access type at initiation of dialysis, we only included those patients still on hemodialysis at 90 days, which we felt strengthened our analysis because it removed the acute dialysis starts who recover kidney function or die in the first 90 days, and chronic dialysis patients who convert to peritoneal dialysis or undergo renal transplantation within the first 90 days. Although this may introduce survival bias, it would be less likely to overestimate the catheter patients who may have started dialysis with a CVC for a well-defined reason (eg/acute unexpected renal deterioration in someone planning PD as definitive renal replacement modality).

There are some limitations to our study. Distance to centre was determined by postal code, and may not reflect true travel distance or the degree of isolation. For example, two communities located the same distance from a dialysis centre may vary widely in that one might be a fly-in community while the other has a well-established road system. In addition, our analysis calculated distance from postal code to closest dialysis centre, which for some patients may have differed from the actual dialysis centre of first hemodialysis. Since the cutoff for furthest distance was > 20 km, there is likely significant heterogeneity in this population. The availability of health care resources may vary widely in that some individuals may have access to hospitals, specialists, and imaging services while others may only have access to a nursing station. Our study does not account for factors related directly to vascular access creation such as surgical assessment, operating room wait times, patient decision, patient suitability for AVF/G creation, and history of failed AVF/G. Since low rates of AVF creation, AVF with delayed maturation and failure to mature are all possible explanations contributing to our results, access creation rate would have been valuable but this information is not available in the CORR database. Furthermore, distance to dialysis centre may not necessarily correlate with distance to pre-dialysis care, where the referral and/or surgical creation of an AVF/AVG would occur. Our data also does not account for patient mobility, where a patient may have relocated from a more remote location to one that is in closer proximity to their nephrology care, thus potentially introducing error in the distance classification for those patients. Lastly, our results could have been confounded by other unmeasured factors (e.g. socioeconomic) that are not collected in the CORR database.

## Conclusions

In summary, we found that increasing residential distance from a dialysis centre was associated with increased use of CVC in incident dialysis patients. This association was mitigated but not eliminated by exposure to adequate pre-dialysis care. Additional efforts to address geographic disparities in dialysis care are needed.

## Competing interests

All authors declared that they have no competing interests.

## Authors’ contributions

MMS and LMM designed the study and drafted the manuscript. MMS managed the database, and performed statistical analysis. JM, CR, and NT assisted in data analysis and editing/revision of the manuscript. CL, LM, PK, and LV were involved in editing and revision of the manuscript. All authors read and approved the final manuscript.

## Pre-publication history

The pre-publication history for this paper can be accessed here:

http://www.biomedcentral.com/1471-2369/15/40/prepub
